# Congenital pseudarthrosis of the proximal tibia: a case report

**DOI:** 10.3389/fped.2026.1768501

**Published:** 2026-01-30

**Authors:** Jiewei Weng, Jiaqi Wang, Tianyou Li

**Affiliations:** Department of Pediatric Orthopedics, Shandong Provincial Hospital Affiliated to Shandong First Medical University, Jinan, Shandong, China

**Keywords:** children, congenital pseudarthrosis of the tibia, proximal CPT, proximal tibial dysplasia, surgery

## Abstract

Congenital pseudarthrosis of the tibia (CPT) is a rare and challenging pediatric orthopedic disorder that predominantly affects the distal or middle third of the tibia, with involvement of the proximal third being exceedingly rare. Literature on this condition remains limited. This case presents proximal tibial CPT with proximal tibial dysplasia in a 7-year-old male patient with neurofibromatosis type 1. Based on the fundamental treatment principles of CPT, the child underwent pseudarthrosis resection, bone grafting, and internal fixation with a Rush rod combined with a plate. Following the achievement of initial successful union, hemiepiphysiodesis was employed to correct the genu valgus deformity. This case report proposes a hypothesis regarding the formation mechanism of proximal tibial CPT and provides clinical support for the standardized diagnosis and management of CPT.

## 1 Introduction

Congenital pseudarthrosis of the tibia (CPT) is a rare and challenging pediatric orthopedic disorder, with an estimated incidence of 1 in 140,000 to 250,000 live births. Approximately 50% of cases are associated with neurofibromatosis type 1 (NF1) (1). The condition is characterized by segmental tibial dysplasia present at birth, or postnatal progressive anterolateral bowing with cystic lesion or narrow medullary canal, which may progress to pathological fracture following minimal trauma or spontaneously, ultimately resulting in pseudarthrosis (1, 2). CPT predominantly affects the middle/distal tibial diaphysis, proximal third involvement remains exceptionally rare, with limited documented cases globally (3, 4). Proximal tibial dysplasia, first described by Cho et al. (5) in 2007, typically radiologically manifests as trumpet-shaped narrowing, anterior inclination, and concavity of the anterior cortex of the proximal tibial physis in cases of middle or distal CPT. He and subsequent researchers demonstrated that distraction osteogenesis at dysplastic proximal segments resulted in prolonged bony consolidation periods and compromised regenerate callus quality (6). However, to date, no studies have reported proximal tibial CPT complicated by proximal tibial dysplasia, nor have they explored the potential association between the two conditions. We report a unique case of proximal tibial CPT with concomitant developmental dysplasia of the proximal tibia in a pediatric patient with NF1, managed at Shandong Provincial Hospital Affiliated to Shandong First Medical University. Institutional review board approval (SWYX:2025-142) and parental informed consent were obtained.

## 2 Case presentation

**Figure 1:**
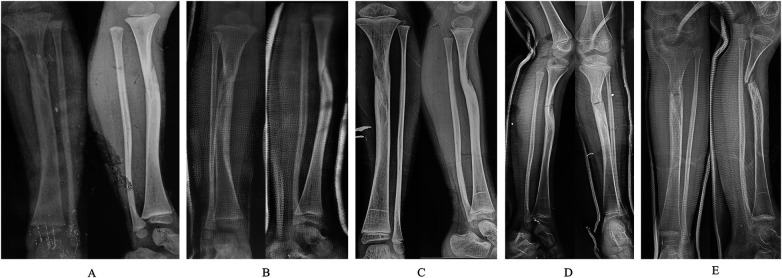
Serial radiographs of the left tibia and fibula before hospitalization. **(A)** On June 7, 2020, radiographs showed initial fracture when the patient was 3 years and 10 months old. **(B)** On July 22, 2020, proximal tibial dysplasia was obvious. **(C)** In January 2022, radiographs demonstrated incomplete fracture union. **(D)** Refracture occurred three months before admission. **(E)** Radiographs 1 month prior to admission showed non-union and pseudarthrosis formation.

On October 29, 2023, a 7-year-old male patient was admitted with a chief complaint of progressive left lower leg deformity over a 3-year period following trauma. On June 7, 2020, the child presented swelling, pain, deformity, and restricted motion in the proximal left lower leg secondary to a fall. Radiographs from a local hospital showed a non-displaced proximal tibial fracture, but the mild anterolateral bowing, dense medullary canal, and cortical thickening went unrecognized, so CPT was not considered (Figure 1A). Treatment was limited to plaster immobilization. Follow-up films on 18 June 2020 revealed the pre-existing anterolateral bowing plus an anterior cortical concavity consistent with proximal tibial dysplasia (5) and marked medullary narrowing from cortical thickening (Figure 1B). At the 18-month review in January 2022, imaging confirmed non-union of the anterior cortex of the dysplastic proximal tibia with further medullary obliteration (Figure 1C). The child continued to walk independently without additional intervention.

Three months prior to admission, minor trauma reactivated leg pain, further deformity, and dysfunction. Radiographs demonstrated a proximal tibial refracture with aggravated concavity of the anterior cortex (Figure 1D). Temporary plaster fixation was applied. Radiographs obtained one month earlier showed no fracture union, bone atrophy, trumpet-shaped medullary narrowing (5) at the fracture ends consistent with proximal tibial dysplasia, and obvious posterior angulation at the non-union site (Figure 1E). After consultations at several hospitals, the patient was referred to our institution. He had been diagnosed with genetically confirmed NF1 three years earlier; his father also has NF1. There was no history of previous surgery, fever, allergy, infection, or developmental delay.

### 2.1 Physical examination

**Figure 2:**
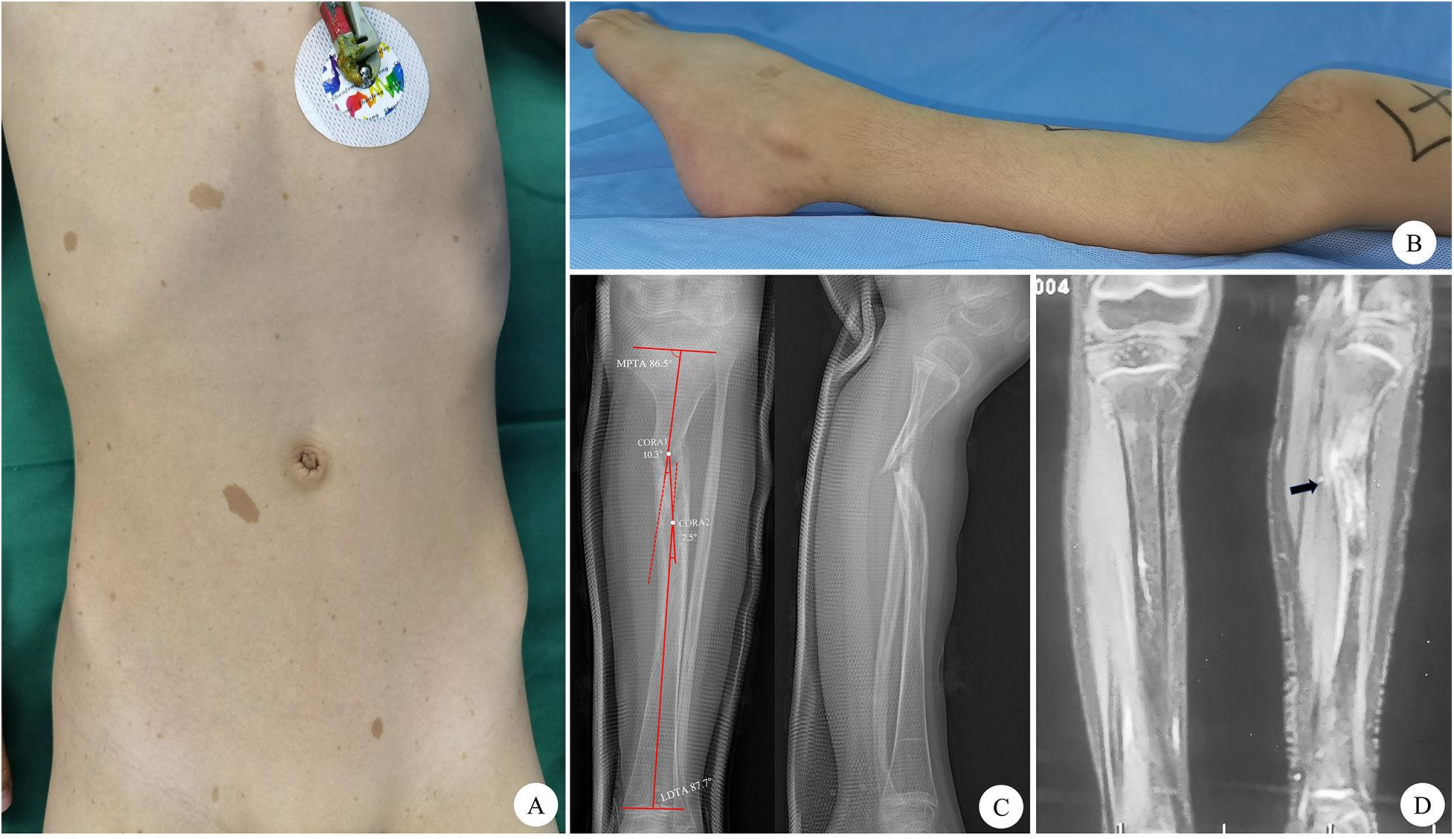
Preoperative physical examination and imaging findings of the child. **(A)** Café-au-lait macules visible on the chest and abdomen. **(B)** Posterior angulation deformity of the proximal left lower leg. **(C)** Preoperative anteroposterior and lateral radiographs demonstrate a pseudarthrosis in the proximal third of the tibia, associated with characteristic proximal tibial dysplasia, manifesting as trumpet-shaped narrowing. Bone atrophy and sclerosis at the bone ends of the pseudarthrosis are also evident. **(D)** T2-weighted MRI of both lower limbs, with black arrows highlighting abnormally high signal intensity in portions of the tissue surrounding the pseudarthrosis.

Multiple café-au-lait spots of varying sizes and shapes were observed across the entire body (Figure 2A). The left lower limb was approximately 1 cm shorter than the contralateral side. The upper 1/3 of the left lower leg exhibited a posterior angulation deformity (Figure 2B), with no local tenderness but abnormal movement noted. Muscle strength and tone of both lower limbs were within normal limits; toe movement, peripheral blood supply, and skin sensation were also normal.

### 2.2 Adjunct examinations

Radiographs showed pseudarthrosis in the proximal 1/3 of the left tibia, characterized by medial and posterior angulation of the sclerotic and atrophic bone ends, along with trumpet-shaped narrowing or even obliteration of the medullary canal. The middle segment of the tibia exhibited anterolateral bowing, resulting in an approximate “S”-shaped deformity of the tibial diaphysis on both anteroposterior and lateral views, with two centers of rotation of angulation (CORA) (Figure 2C). Measurements indicated a medial proximal tibial angle (MPTA) of 86.5°, a lateral distal tibial angle (LDTA) of 87.7°, CORA1 angle of 10.3°, and a CORA2 angle of 7.5° (Figure 2C). Magnetic resonance imaging (MRI) confirmed pseudarthrosis formation in the proximal left tibia, with cortical bone discontinuity. Abnormal high signal intensity was observed on T2-weighted imaging within the pseudarthrosis space, which was interpreted as being filled with fibrous and cartilaginous tissues (Figure 2D). Genetic testing identified a heterozygous mutation in the NF1 gene (c.3315-3C>G), leading to a splice mutation affecting the corresponding amino acid sequence.

### 2.3 Diagnosis

Based on the history, physical examination, and adjunct examinations, the child was diagnosed with: (1) Congenital proximal tibial pseudarthrosis, specifically the atrophic Crawford IV type; (2) NF1.

### 2.4 Surgery

**Figure 3:**
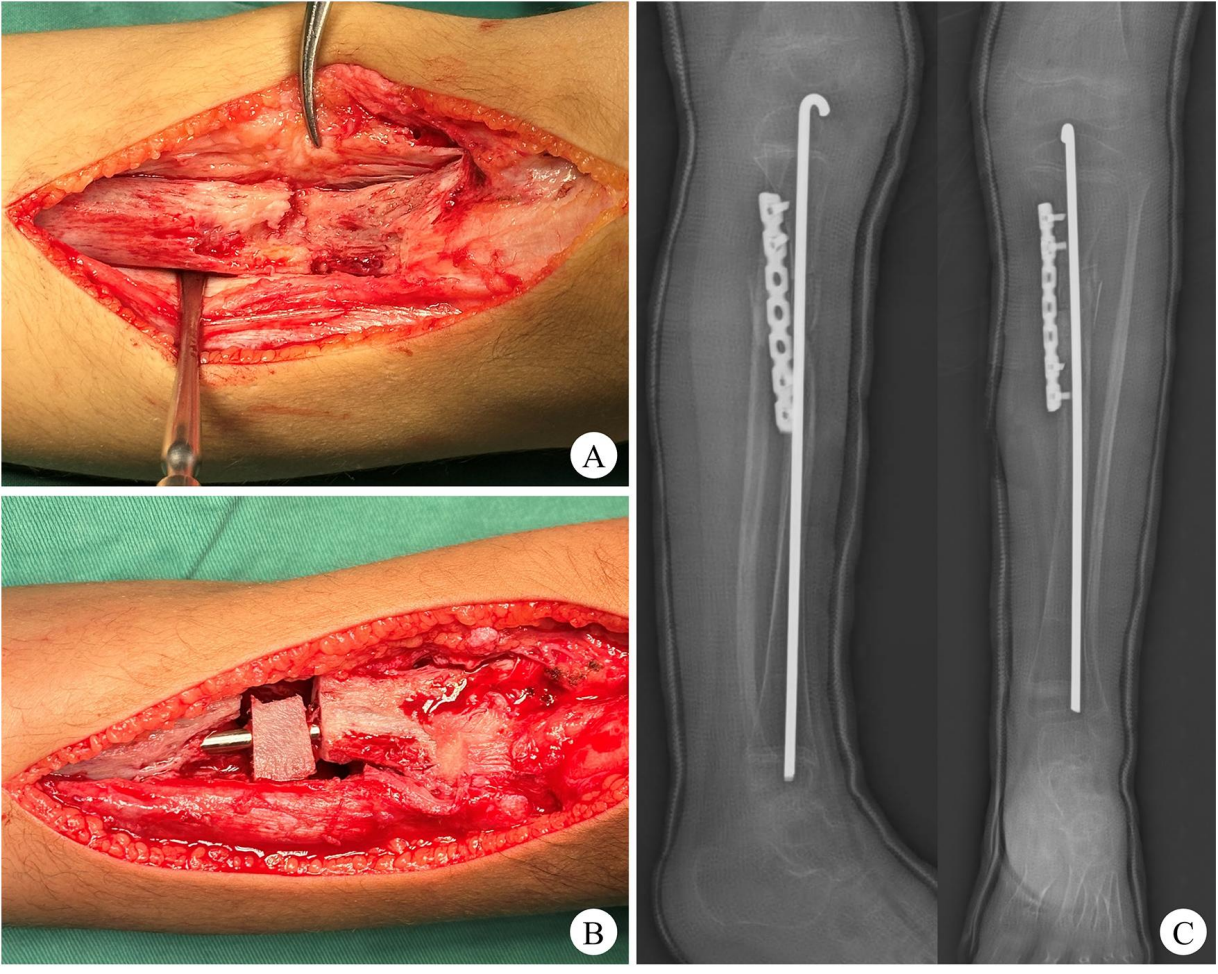
Intraoperative photographs and two-day postoperative radiographs of the tibia. **(A)** Localized bony dysplasia at the pseudarthrosis site with pathologically thickened fibrous periosteum. **(B)** The Rush rod sequentially traverses the proximal tibia, iliac bone graft, and distal tibia. **(C)** Satisfactory reduction (with proper alignment and position) of the osteotomy ends and optimal placement of the internal fixation.

The indications were definitive. The procedure involved resection of the left tibial pseudarthrosis and surrounding tissue, followed by autologous iliac bone grafting, periosteal transplantation, and internal fixation using a Rush rod and a locking compression plate (LCP). Under general anesthesia, the left lower limb and hip underwent conventional sterilization and draping. A 12-cm longitudinal incision was made over the anterior left tibia, extending from the proximal tibial tuberosity to 5 cm distal to the pseudarthrosis site. The subcutaneous tissues were dissected to expose the pseudarthrosis and anterior tibial plateau. Intraoperatively, the pseudarthrosis was surrounded by hyperplastic, thickened periosteum; the adjacent bone was hypoplastic and atrophic, with a whitish discoloration, and the bone ends were bridged by fibrous tissue (Figure 3A). The pseudarthrosis was excised until bleeding bony edges were obtained. The pathologically hyperplastic periosteum was resected and submitted for histopathological examination. The medullary canal was reamed with a drill, revealing a 2 cm bone defect. A 4 cm curved incision along the iliac crest exposed the outer iliac cortex, allowing harvest of a full cortico-cancellous graft measuring 3 × 2 × 1 cm. Additional iliac cortical strips (2.0 × 0.5 cm), cancellous bone, and periosteal grafts (3 × 4 cm) were also collected. A 4.0 mm Rush rod was inserted through a drill-create entry hole at the anterior tibial plateau, traversing the proximal tibia, iliac bone block, and distal tibia (Figure 3B). Under fluoroscopic guidance, lower limb alignment was optimized, and the LCP was applied to stabilize the osteotomy site. Residual bone grafts were packed around the defect, and the periosteum was suspended and sutured to the surrounding normal tissues. The wound was irrigated and closed in layers. A long-leg cast was applied postoperatively.

### 2.5 Follow-up

**Figure 4:**
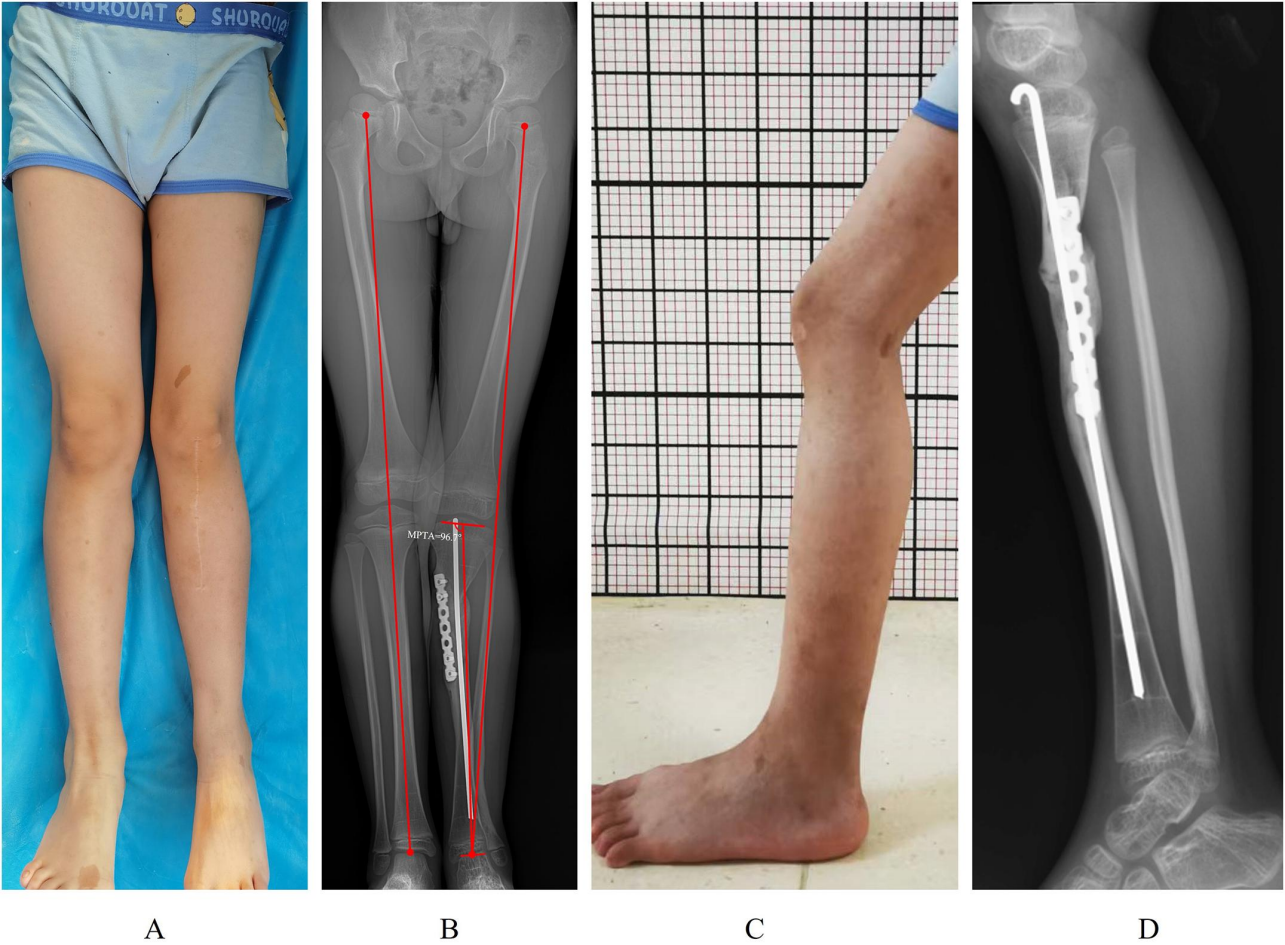
Lower limb appearance and radiographs of the child at final follow-up. **(A)** Anterior clinical photograph showing equal limb lengths and mild left genu valgum. **(B)** Full-length anteroposterior radiograph of both lower limbs showing left genu valgum and 0.6 cm of left-sided shortening. **(C)** Lateral clinical photograph demonstrating normal morphology of the left leg.

**Figure 5:**
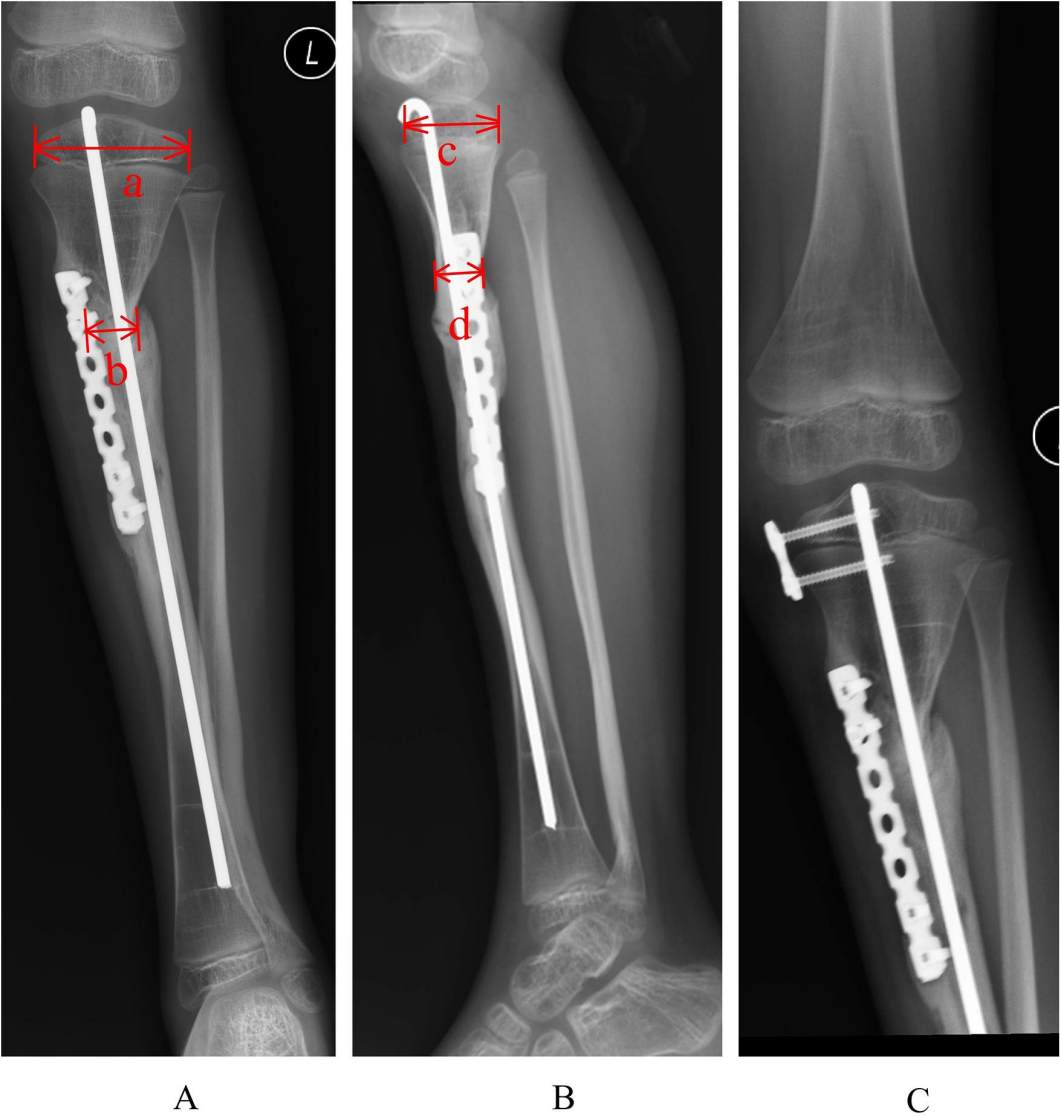
Refracture risk evaluation via anteroposterior and lateral radiographs. The cross-sectional area of the union region is calculated as the product of the narrowest diameter of the pseudarthrosis union on the AP radiograph **(A)** (denoted as b) and the narrowest diameter on the lateral radiograph **(B)** (denoted as d). The cross-sectional area of the proximal tibial epiphysis is determined by multiplying the widest diameter of the epiphysis on the AP radiograph **(A)** (denoted as a) by the widest diameter on the lateral radiograph **(B)** (denoted as c). The relative cross-sectional area is defined as (b × d)/(a × c).

Postoperative 2-day radiographs confirmed appropriate alignment, adequate graft placement, and satisfactory internal fixation positioning (Figure 3C). The cast was removed at 6 weeks, after which partial weight-bearing was initiated. At final follow-up (20 months postoperatively), the patient ambulated independently without joint pain but exhibited mild gait asymmetry. Surgical scars on the left hip and anterior tibia were well-healed. The right lower limb was approximately 0.6 cm longer than the left, with mild valgus at the left knee (Figures 4A,B). The range of knee and ankle motion remained unrestricted. Radiographs demonstrated abundant callus formation along the posterior and lateral cortices at the pseudarthrosis site, while the medial and anterior cortices remained contiguous with faint residual radiolucency (Figures 4C,D). The modified Radiographic Union Score for Tibial fractures (RUST) score was 10/12 according to Richard' s criteria (7), indicating initial union. The MPTA measured 96.7°, suggesting that the genu valgum deformity was primarily caused by proximal tibial valgus. Per Choi' s method (8), the cross-sectional area (CSA) of the union site was 290 mm^2^, yielding a relative CSA of 0.16 (Figures 5A,B). At final follow-up, temporary medial hemiepiphysiodesis of the proximal tibia was performed to achieve gradual correction of the valgus deformity. (Figure 5C).

## 3 Discussion

CPT primarily affects the middle to distal regions of the tibia, with involvement of the proximal third being exceedingly rare. A European multicenter study (9) reported incidence rates of 56% for distal, 42% for middle, and 2% for proximal CPT, noting that lesion location may undergo minor shifts during growth. Yang et al. (3) observed proximal-third CPT in only 14 out of 497 cases (2.8%) over a decade. Current literature lacks detailed characterization of proximal CPT pathology and associated treatment strategies. Khiami et al. (4) documented a single case followed to skeletal maturity, in which severe bone loss and 12 cm limb shortening persisted despite multiple surgical interventions, ultimately necessitating amputation.

The etiology and pathogenic mechanism of CPT remain poorly understood. Micro-pathological anatomy studies indicated that the bone-remodeling imbalance in CPT is critically driven by an annular stenosis of fibrous hamartomatous tissue at the pseudarthrosis site and by an abnormally thickened, fibrotic periosteum (2). Additionally, bone malnutrition (stemming from periosteal vascularization defects and reduced osteogenic capabilities) further exacerbates this process (10). Cho (5) noted that the periosteum overlying the dysplastic proximal tibia is consistently thickened and markedly fibrotic, which is consistent with pathological changes observed in lesioned periosteum or proliferative fibrous hamartomatous tissue surrounding the distal CPT. The abnormal tissue extent varies among different patients, and some even exhibit extensive involvement extending to the proximal tibial metaphysis. In addition to the consistency of periosteal lesions, the pathological changes and prognoses of the dysplastic proximal tibia after osteotomy are also similar to those of the distal CPT bone ends. The same study (5) found that the dysplastic tibial segment displays a significantly narrowed cross-sectional area at the corticotomy site. Following distraction osteogenesis, bone regeneration at the corticotomy site is impaired, with abnormal regenerate callus morphology and a prolonged healing index of up to 117 days/cm, which aligns with poor bone healing capacity at pseudarthrosis sites (5, 6).

Preoperative serial radiographs showed mild anterolateral tibial bowing and, more importantly, established proximal tibial dysplasia that antedated the subsequent pseudarthrosis. Typical manifestations included trumpet-shaped stenosis of the proximal tibia and anterior cortical concavity, with no significant abnormalities noted in the distal tibia. Intraoperatively, the proximal pseudarthrosis site exhibited a lesioned periosteum with marked fibrous hyperplasia and hypertrophy. Therefore, based on pathological similarity, the authors believe that the dysplasia of the proximal tibia is similar to the pathological changes that occur in the pre-pseudarthrosis stage of the distal CPT, such as tibial bowing, medullary cavity stenosis and obliteration. These alterations render the proximal tibia highly susceptible to fracture; once fractured, the ends fail to unite spontaneously, evolving into pseudarthrosis that angulates posteriorly and medially. However, the reason for the greater predilection of CPT for the distal tibia over the proximal tibia remains unclear.

The primary treatment for CPT is surgery, aimed at achieving long-term bone union, preventing refracture, and avoiding lower limb mechanical axis deviation, lower limb length discrepancy, and adjacent joint stiffness. Established techniques include combined bone grafting with intramedullary (IM) nails and external fixation, vascularized fibular graft transplantation, the Masquelet technique, and the Cross-Union, etc. Despite diverse approaches, core principles remain consistent: 1. Resection of sclerotic pseudarthrosis, pathologic periosteum and fibrohamartomatous tissue; 2. Structural bone grafting at the CPT site; 3. Optimized stabilization (11, 12). Currently, the combined bone grafting-IM nails-external fixation approach is most commonly adopted in clinical practice. Numerous studies have demonstrated that this method achieves superior initial union and lower refracture rates in contemporary practice (11–13). However, CPT cases involving the proximal tibia are relatively rare, leading to a lack of specific surgical experience. Drawing on experience from distal CPT treatment, this case employed pseudarthrosis resection, autologous corticocancellous iliac grafting, periosteal transplantation, and fixation with a Rush rod and LCP. Compared to the commonly used Ilizarov external fixator in combined procedure, plating provides easier postoperative care, improved patient comfort, and a reduced infection risk. In 2012, Paley et al. described the cross-union technique for CPT treatment, refining it in 2017 by replacing the Ilizarov frame with a malleable LCP, resulting in a 100% union rate without refracture (14). Paley and recently published studies emphasized that both plating and external fixation effectively compress the CPT pseudarthrosis site and control rotation (14, 15). Initial union was achieved in this case and no refracture has occurred to date. If persistent bone nonunion occurs, the vascularized tibial periosteal graft technique proposed by Soldado would be considered for implementation under the guidance from department of microsurgery (16). If the intractable bone nonunion persists despite interventions, we may ultimately choose the below-knee amputation referenced by Khiami (4).

In distal CPT, rigid fixation of the distal fragment typically requires inserting the IM nail through the heel, traversing the tibiotalar and subtalar joints. However, such across-ankle IM nailing may lead to reduced ankle range of motion and even ankle stiffness (11–13). With growth, the IM nail head may retract into the metaphysis, potentially reducing the fixation range in distal CPT and thus making a lengthening IM nail a preferable alternative. In the case, as the lesion was proximal, the Rush rod was inserted via the tibial plateau and fixed into the distal tibial epiphysis without violating the ankle joint, so ankle joint function remained intact. Whether the subsequent retraction of the rod into the distal metaphysis increases the refracture risk will require continued surveillance. Some researchers have noted that a larger CSA at the pseudarthrosis site and a greater relative union area correlate with extended fracture-free survival (8, 17). Specifically, the research data from Choi (8) demonstrated that in the high-risk refracture group, CSA was (142.8 ± 37.64) mm^2^ and relative cross-sectional area was (0.13 ± 0.04). Compared to these values, this child's results suggest both factors may heighten refracture risk necessitating long-term close follow-up.

Abnormal lower limb alignment is observed during the treatment of CPT, with proximal tibial valgus being a relatively common complication (18). The exact cause of this complication remains unclear; however, some scholars hypothesize that proximal tibial dysplasia may lead to uneven growth of the proximal physis, resulting in proximal tibial valgus (13). In the case, the left proximal tibia demonstrated approximately 6.7° of valgus (Figure 4B). This finding may be associated with poor lateral union at the pseudarthrosis site, and the possibility of proximal tibial dysplasia can't be excluded. According to research by Deng et al. (18), elective surgery for alignment correction is indicated. At the final follow-up, the child underwent temporary medial hemiepiphysiodesis of the proximal tibia (Figure 4C). Besides the ongoing correction of genu valgum deformity (i.e., proximal tibial valgus), the child remains at risk of ankle valgus in the future, if so, medial hemiepiphyseal block of the distal tibia will be performed. At the latest follow-up, the child had only mild shortening of the left lower limb relative to the right (0.6 cm), and conservative treatment such as shoe lift padding can be adopted currently. If the extent of limb length discrepancy increases larger than 2 cm, no form of distraction osteogenesis on the affected limb will be considered, arresting the growth of the contralateral leg may be acceptable in the case.

Regarding the iliac bone graft technique, a corticocancellous bone block was harvested from the ilium to function as a structural graft for bridging support. Compared to cancellous bone or allograft, these grafts offer superior resistance to resorption and can help avoid the premature development of lower limb length discrepancy (1). At the last follow-up, initial union of the pseudarthrosis was successfully achieved. However, compared to wrapping or onlay bone grafting techniques, the efficacy of corticocancellous bone grafting necessitates validation through additional CPT cases (13). Notably, influenced by the focus on periosteal transplantation in Paley's early work and subsequent studies, an autologous iliac periosteal graft was also transplanted to the pseudarthrosis site in the case (14).

Genetic testing confirmed a diagnosis of NF1 in the child, revealing a heterozygous mutation (c.3315-3C>G) in the NF1 gene. Data indicates that approximately 50% of children with CPT have NF1 (2), though this proportion may be underestimated; a systematic review by Kjell in 2016 reported an actual prevalence as high as 84% (19). NF1 is an autosomal dominant disorder caused by mutations in the NF1 gene, which lead to loss of neurofibromin function. This dysfunction dysregulates signaling pathways such as Ras/mitogen-activated protein kinase, ultimately inhibiting osteoblast differentiation and enhancing osteoclast activity at the pseudarthrosis site (2). Based on this mechanism and clinical trial data, some researchers regard NF1 as a negative predictive factor for CPT union (20). However, recent studies challenge this perspective. Borzunov et al. (21) found equivalent healing rates in CPT cases with and without NF1. Pannier (2) suggests that the pathophysiology of CPT may be identical irrespective of NF1 comorbidity, supported by the identical histological appearance of hamartomatous tissue in both groups. Consequently, whether NF1 impacted union in this CPT case remains uncertain.

## 4 Conclusion

In summary, this report describes the first documented case of proximal CPT accompanied by proximal tibial dysplasia, detailing its clinical presentation, diagnosis, management, and follow-up. The findings lend support to the hypothesis of an association between these two entities and reinforce the value of adhering to established CPT treatment protocols; following these principles, primary bone union was achieved and joint function preserved. Nonetheless, the persistent risks of progressive genu valgum and refracture mandate continued close surveillance. Treatment algorithms for proximal CPT must await broader evidence from larger case series and multicenter studies.

## Data Availability

The raw data supporting the conclusions of this article will be made available by the authors, without undue reservation.
